# Rheology of Alkali-Activated Blended Binder Mixtures

**DOI:** 10.3390/ma14185405

**Published:** 2021-09-18

**Authors:** Biruk Hailu Tekle, Ludwig Hertwig, Klaus Holschemacher

**Affiliations:** Structural Concrete Institute (IfB), Leipzig University of Applied Sciences (HTWK Leipzig), 04277 Leipzig, Germany; ludwig.hertwig@htwk-leipzig.de (L.H.); klaus.holschemacher@htwk-leipzig.de (K.H.)

**Keywords:** rheology, alkali-activated cement, plastic viscosity, yield stress, thixotropy

## Abstract

Alkali-activated cement (AAC) is an alternative cement that has been increasingly studied over the past decades mainly because of its environmental benefits. However, most studies are on heat-cured AACs and are focused on mechanical properties. There is a lack of research on the fresh properties of ambient-cured AAC systems. This study investigates the rheological properties of ambient-temperature-cured alkali-activated blended binder mixtures activated with sodium silicate and sodium hydroxide solutions. The influence of binder amount, alkaline solid to binder ratio (AS/B), sodium silicate to sodium hydroxide solids ratio (SS/SH), and total water content to total solid (from the binding materials) ratio (TW/TS) on the rheological properties are investigated. The effect of borax as an admixture and silica fume as a replacement for fly ash is also investigated. The results showed that both the yield stress and plastic viscosity are mainly affected by the binder content and TW/TS ratio decreasing with the increase of each parameter. The yield stress increased with the increase of the SS/SH ratio. Borax significantly reduced the yield stress, while silica fume’s effect was dependent on its dosage.

## 1. Introduction

Concrete is the most commonly used construction material. However, with today’s environmental consciousness and the high carbon dioxide (CO_2_) emission associated with the production of concrete ingredients, especially ordinary portland cement (OPC), this material faces a significant challenge. Alkali-activated cement (AAC) is a relatively new type of alternative cement to replace OPC. It is mainly produced from byproduct or secondary product materials rich in silicon and aluminum such as fly ash (FA) and ground granulated blast furnace slag (GGBS). In the presence of alkaline solutions such as sodium silicate and sodium hydroxides, these materials undergo dissolution. By combining with oxygen and incorporating sodium, potassium, or calcium ions depending on the materials used, the dissolved ions form calcium aluminosilicate hydrate (C-A-S-H) or sodium aluminosilicate hydrate (N-A-S-H) [1]. The main advantage of AAC is its environmental benefits, with an 80% or greater reduction in CO_2_ emission compared to OPC [2,3]. Further to this, AAC concrete has desirable mechanical and durability behaviors such as high strength, good bond performance, excellent fire resistance, and acid resistance [3,4,5,6]. These behaviors have inspired enormous research interest in this alternative binder. 

AAC concrete has been reported to suit various applications ranging from conventional cast-in-place concrete to 3D printing material and more [7]. Certain applications require special attention on the deformation and flow behavior of the concrete, i.e., rheology. For instance, concrete for 3D printing requires high yield stress at rest and low plastic viscosity during flow [8]. The rheological properties of the 3D printing concrete can be used for achieving a balance between pumpability, extrudability, and buildability [9]. Early concrete processing, such as spreading, molding, and compaction, depends on its rheological properties.

Interest in the rheological behaviors of AAC systems is increasing as the mortar and concrete based on this binder are considered for various applications [10,11,12]. The fresh AAC mixture is a solid-liquid suspension system and its rheology depends on various parameters such as type and amount of source materials and activators used, chemical admixtures, temperature, solid contents, water content, and so on [12,13,14,15,16]. Ground granulated blast furnace slag (GGBS), fly ash (FA), and silica fume (SF) are some of the commonly used source materials. Sodium silicate and sodium hydroxide, mixed or separately, are the most common activators used. Using GGBS enhances AAC mixtures’ ambient curing behavior [17]. This is due to the high calcium oxide content and fineness of GGBS, improving the binder’s reactivity and enhancing the reaction product formation at ambient curing conditions. However, the same reason also reduces workability and affects the rheology [18,19,20]. Sodium hydroxide or a combination of sodium hydroxide and sodium-silicate-activated AACs follows the Bingham model [11,21], meaning the relation between shear stress and shear rate is linear, while sodium-silicate-activated AACs follow the Herschel–Bulkley model [16], i.e., a nonlinear relation between shear stress and shear rate.

Achieving desired rheological behaviors is relatively easy in OPC concrete as various admixtures are available. However, admixture technologies are still in development for AAC concrete. Palacios et al. [22] investigated the stability of different superplasticizers (polycarboxylate, vinyl copolymer, melamine, and naphthalene derivatives) in water and different alkaline solutions. All the superplasticizers except the naphthalene-based one are unstable at high pH (greater than 13). Tong et al. [23] stated that there are almost no superplasticizers that can improve the workability of sodium-silicate-activated slag, showing that the performance of the superplasticizers is also dependent on the alkaline activators. Hence, developing the required rheological behaviors of AAC systems has a critical challenge.

In this study, the rheological behavior of AAC is studied. Different mix design factors such as water to binder ratio, sodium silicate to hydroxide ratio, activator to binder ratio, and binder content are studied for their effect on rheology. Furthermore, the effect of SF, borax (a potential set retarder), and a new type of superplasticizer is also investigated.

## 2. Experimental Details

### 2.1. Materials

**Figure 1:**
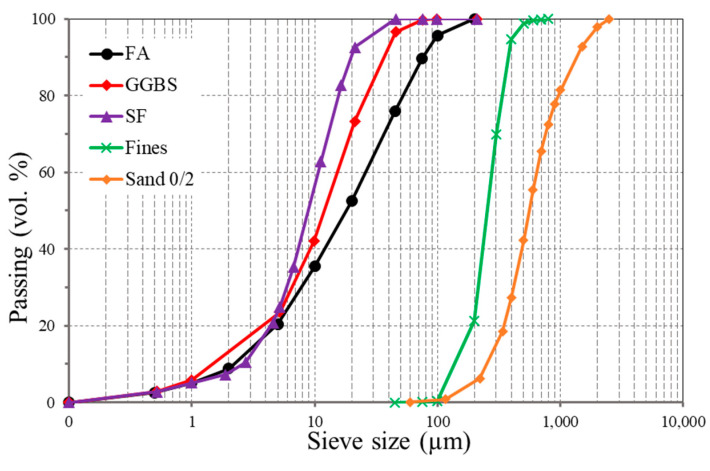
Particle size distribution.

**Figure 2:**
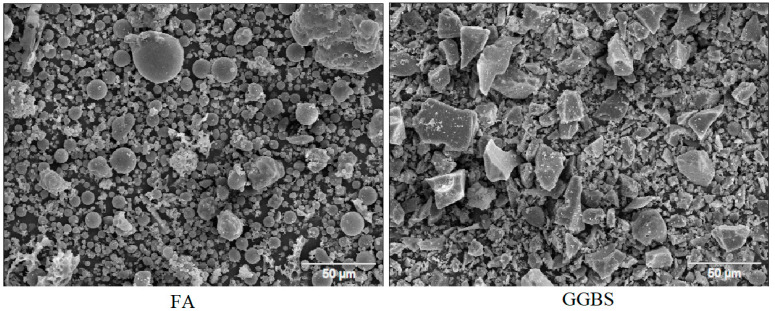
SEM image of source materials.

FA, GGBS, and SF were used in the preparation of the alkali-activated concretes. The FA, GGBS, and SF comply with the EN 450-1 [24], EN 15167-1 [25], and EN 13263-1 [26] requirements, respectively. The chemical compositions of these source materials are summarized in Table 1. The chemical analysis was performed using energy dispersive X-Ray analysis (EDX) (Noran System SIX) and a scanning electron microscope (Jeol JSM–IT 100, JEOL Ltd., Freising, Germany). Figure 1 shows the grain size distributions for each ingredient obtained by laser diffraction method. The activator solution used is a mixture of sodium silicate and sodium hydroxide. The sodium silicate solution includes 26.82% silicate, 8.2% sodium oxide, and 64.98% water, while the sodium hydroxide is a 50% by weight solution. New superplasticizer underdevelopment (Geo-1) supplied by Sika Germany was used. Furthermore, sand with a maximum aggregate size of 2 mm (Sand 0/2 in Figure 1) was used. Additional fines (shown in Figure 1 as Fines) were also used at 10% of total sand. The chemical composition of the binders is shown in Table 1. Figure 2 shows the morphology of FA and GGBS observed by scanning electron microscope (SEM). The FA particles are spherical, and the GGBS particles have an irregular shape with high angularity.

### 2.2. Investigated Parameters and Mix Proportions

The research is part of an ongoing project for developing a concrete mixture for use in textile-reinforced concrete. Textile reinforcement requires fine-grained concretes due to their smaller openings; hence, no coarse aggregate was used. The constituents of the precursor were set at 55% FA, 40% GGBS, and 5% SF based on Tekle et al. [28]. Three mixes were designed targeting a medium- and two high-strength classes (AAC-1, AAC-2, and AAC-3). Their respective 28 days strengths were 40 MPa, 72 MPa, and 92 MPa. The mix parameters of AAC-2 were then varied to understand their effect on the rheology of the mixture. The parameters were binder (precursors) content (B), alkaline solid to binder ratio (AS/B), sodium silicate to sodium hydroxide solids ratio (SS/SH), and total water to total solid binders ratio (TW/TS). TW is the amount of free water and water from the alkaline solution, while TS is the total amount of solid binding material in the mixture, i.e., B and AS. AAC-2 was taken as a reference mix, as shown in Table 2.

### 2.3. Test Methods and Specimen Preparation

The sodium silicate and sodium hydroxide solution were mixed in the required proportion at least 24 h before mixing. The specimens were prepared by first mixing the dry materials (sand and binder) in a mixer for about two minutes. Afterward, the prepared alkaline solution was mixed with the additional water, added slowly to the dry mixture, and mixed for about four minutes. In the case of mixes with superplasticizer (SP), the SP was added halfway during the wet mixing.

**Figure 3:**
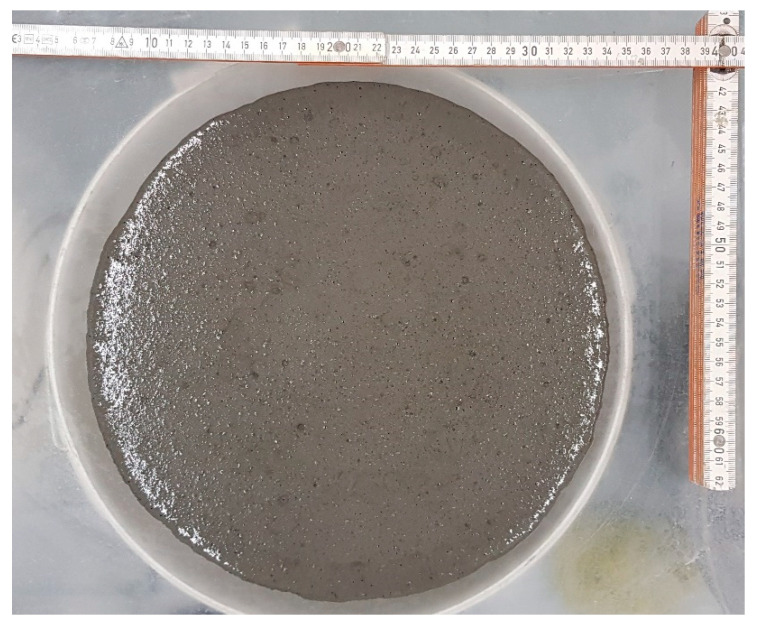
AS/B.18 mix during flow test.

**Figure 4:**
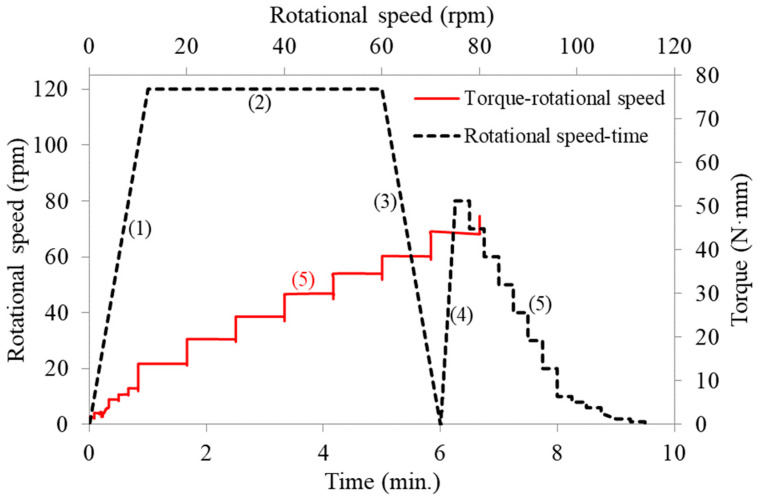
Typical torque–rotational speed relationship (AS/B.18).

The consistency of the fresh mixture is determined by flow tests according to EN 1015-3 [29]; however, without the jolting, as the mixes were relatively workable. Figure 3 shows AS/B.18 mix during the flow test. The fresh mixture was then removed from the flow table and added back to the mixing bowl and mixed for an additional 15 s. The rheology behavior of the mixtures was tested using a rotational viscometer (Viskomat NT) equipped with a cylindrical container of 100 mm in height and 83 mm in diameter. The paddle consists of four symmetrically arranged curved rods; a fishbone paddle. The test was carried out about 10 min after the addition of water into the dry ingredients. The rotational speed was ramped from 0 to 120 rpm in 1 min (step 1 in Figure 4). It was kept at 120 rpm for 4 min. Then, the speed was ramped down to 0. The rotational speed was then ramped up again from 0 to 80 rpm and ramped down in steps. The torque under each imposed speed was measured. The rheology parameters, yield stress, and plastic viscosity were then obtained according to the torque–rotational speed relationship.

## 3. Results and Discussion

### 3.1. Results

The rheology of AAC is affected by the type of activator and the type of precursor used [21,30]. In Na_2_SiO_3_-activated systems, the Herschel–Bulkley model is a better fit [21]. In NaOH-activated systems, Bingham model was reported to fit better, meaning that their rheology is defined by two physical parameters, i.e., yield stress and plastic viscosity [21]. The AAC in this study uses both these activators. Figure 4 shows the five steps used for the flow profile and the torque–rotational speed relationship for the last step (stepwise descending part) of the flow curve. The torque increased and decreased with the respective increase and decrease of the rotational speed. For the evaluation of the rheological properties, the data points of the downward stepwise ramp (step 5) were selected. The duration of each of the steps in the downward curve is 15 s (Figure 4). A linear regression of the torque and the rotational speed in the 20–80 rpm range was performed. The regression lines showed a linear relation, showing that the structure is broken down to equilibrium by the applied shear stress during the test cycle. This behavior was observed in all the mixes, showing that the relationship between the shear stress (τ) and the shear rate ($\dot{\gamma }$) for the studied AAC mixture can be described using the Bingham model, as shown in Equation (1).

(1) $$\tau =\tau_{0}+\eta \dot{\gamma }$$

where $\tau_{0}$ is the yield stress, and η is the plastic viscosity.

The data from the Viskomat in the current study are for torque and rotational speed. Such data are only relative values as they are geometry-specific. Converting them to shear stress and shear rate is complex, especially with probes such as the one used in the current study (fishbone). Haist et al. [31] conducted an interlaboratory study for different geometries and recommended conversion factors for absolute value shear stress and shear rate determination based on the affine-translation approach. In the current study, the yield stress and plastic viscosity values were obtained by using these conversion factors. The flow values and the yield stress and viscosity parameters for each of the mixes are summarized in Table 3.

### 3.2. Flowability

**Figure 5:**
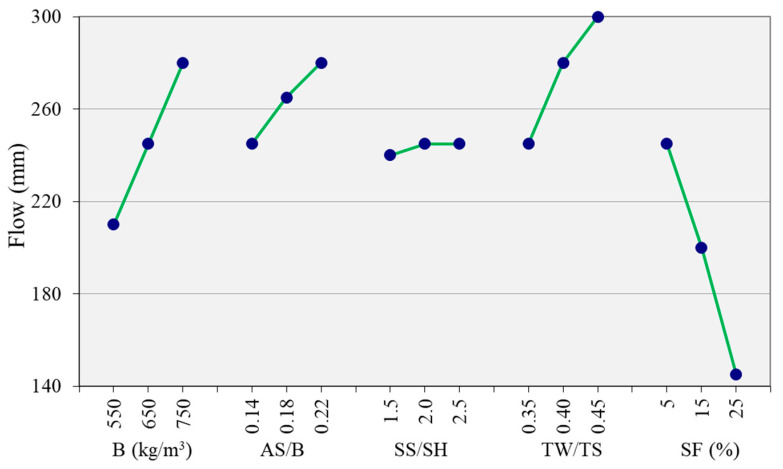
Effect of parameters on the flow.

Figure 5 shows the main effect plots for the flow values of the mix proportion parameters studied. All the investigated parameters increased the flow. Binder content and the TW/TS ratio are the most significant factors. The least significant factor is the SS/SH ratio, with a minor increase as the ratio increased. Nath and Sarker [19] observed an opposite effect of sodium silicate to hydroxide solution ratio on the slump and concrete flow. This difference could be due to the difference in source materials used (no SF was used in [19], and the proportion of GGBS and FA is different) or the type of activators used.

The binder content showed a significant effect on the flow. The mixture became more workable as the binder content increased. An increase in binder content by keeping the AS/B and TW/TS ratio constant increased the paste content, ultimately raising the workability. This is consistent with previous study [32].

The AS/B is another significant factor affecting the flowability of the mixture. The flow values increased with the increase of the AS/B. Previous studies reported that the increase in alkaline liquid to binder ratio (AL/B) increased workability [18,19,33]. However, it is not easy to verify if this effect comes solely from the increase in water or the increase in the alkaline solids. It is well known that an increase in water increases workability; however, the current study also confirms that the increase in alkaline solid also increases workability.

The addition of the SP improved the flowability of the mixture from 245 mm flow to 275 mm. Borax also improved the flowability, and the addition of both the borax and the SP did not show additional improvement when compared to the SP-only mixture. The increase in SF from 5% of the binder (control mixture AAC-2) to 15% and 25% reduced the flow value from 245 mm to 200 mm and 145 mm, respectively. This is due to the small particle size, high specific surface area (Table 1), and high reactivity of SF, resulting in higher water requirements and accelerating the formation of flocculants [34,35].

### 3.3. Rheology Results and Analysis

**Figure 6:**
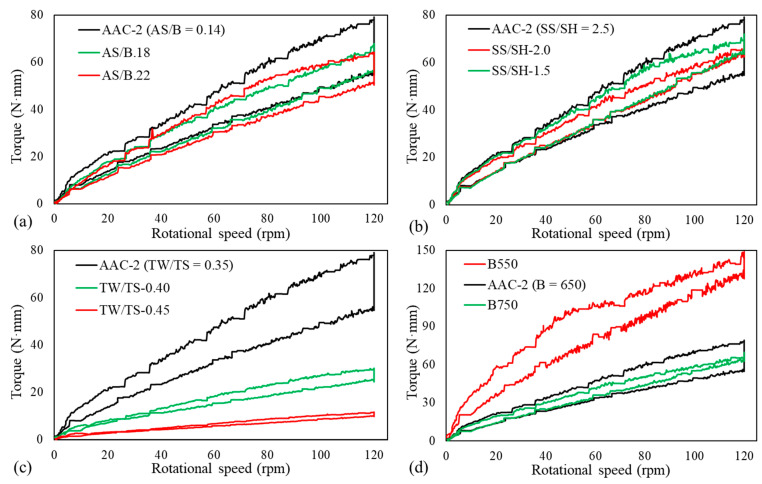
Torque–rotational speed curves for mix proportion parameters: (**a**) AS/B, (**b**) SS/SH, (**c**) TW/TS, (**d**) binder content.

**Figure 7:**
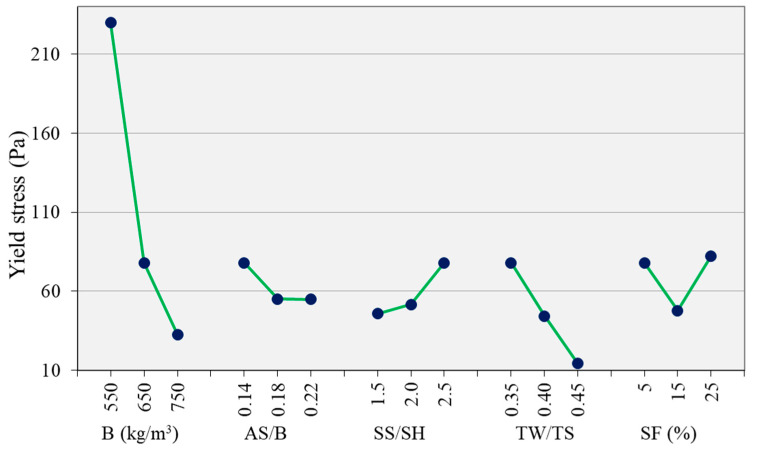
Effect of parameters on the yield stress.

**Figure 8:**
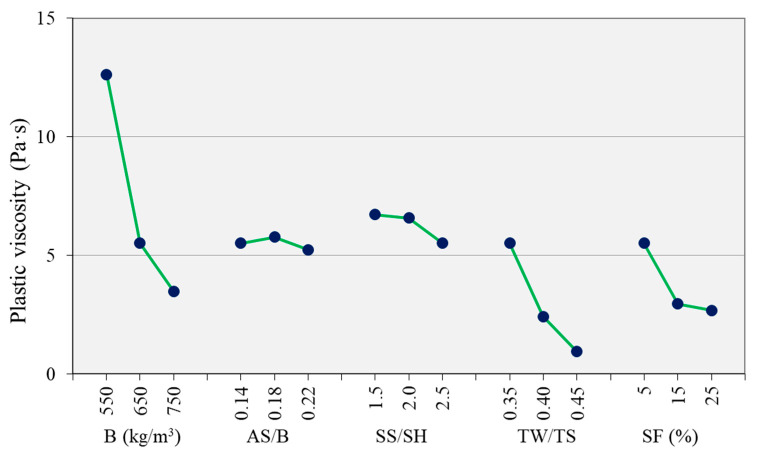
Effect of parameters on the plastic viscosity.

Figure 6 shows the torque–rotational speed curves for the ascending and descending parts for the different mix proportion parameters. The curves show a distinct variation on parameters such as TW/TS and binder content as the parameter is varied. Figure 7 and Figure 8 show the effect of the different parameters on the yield stress and plastic viscosity, respectively. The yield stress and the plastic viscosity mainly show a decreasing trend with the increase of the parameters except SS/SH ratio. The yield stress increased as SS/SH ratio increased. Further discussion on each parameter is presented in the following sections.

#### 3.3.1. Effect of AS/B

The yield stress decreased with the increase of AS/B ratio from 0.14 to 0.22 (Figure 7). A similar effect has been observed by Li et al. [10] when the alkaline content to binder ratio was increased from 0.1 to 0.2 on a one-part alkali-activated slag. Zhang et al. [15] also observed a decrease in both yield stress and plastic viscosity as the alkaline content increased. The amount of microbubbles increases with the increase of alkaline solution [10]. Such microbubbles may decrease the yield stress of the mixture through their ball bearing and lubrication effects. Zhang [15] reported more agglomerations of precursor particles with a lower alkali concentration because of the larger electrostatic force between the particles. This may be one reason for why the yield stress of the mixtures decreased with the increase of AS/B. The plastic viscosity showed no significant change as the AS/B increased (Figure 8).

#### 3.3.2. Effect of SS/SH

The yield stress increased with the SS/SH ratio while the plastic viscosity showed a minor decrease. Palacios et al. [21] reported that the initial yield stress values for Na_2_SiO_3_-activated AAC were higher than the values for NaOH-activated AAC. Hence, the high yield stress in the current study is caused by the higher silicate amount as the SS/SH ratio increased. Increasing the proportion of sodium silicate solution generally encourages gel formation [36]. Palacios et al. [21] and Puertas et al. [30] reported that AAC activated with sodium silicate has a lower setting time than those with sodium hydroxide. This is due to the formation of primary calcium silicate hydrate (C-S-H) gel in the early stages of the reaction due to the bonding of the Ca^2+^ ions from the slag to the silicate ions in the sodium silicate. The faster reaction and formation of reaction products in sodium silicate could be the reason for the higher yield stress observed. The plastic viscosity of the mixtures showed a minor decrease with the increase of the SS/SH ratio.

#### 3.3.3. Effect of TW/TS

Both the mixtures’ yield stress and plastic viscosity decreased with the increase of the TW/TS ratio (Figure 7 and Figure 8). Yield stress and plastic viscosity increase with the increase of solid volume fraction [37]. As TW/TS increases, the solid volume decreases. The distance between solids increase, resulting in a lower particle interaction and hence higher fluidity. The higher fluidity also creates more air bubbles in the mixture. The air bubbles have a ball bearing effect [38]. This reduces the yield stress and the plastic viscosity of the mixtures. Flocculation and, to some extent, reaction product formation are enhanced by a lower TW/TS ratio [39]. Hence, as this ratio increases, the amount of flocs and reaction products becomes lower, resulting in lower rheological parameters. This behavior makes it possible to adjust the rheological behaviors of AAC mixture by adjusting the water content. However, this is not always possible as the water content also controls other essential behaviors such as strength and durability.

#### 3.3.4. Effect of Binder Content

Binder content significantly influenced both yield stress and plastic viscosity (Figure 7 and Figure 8). This is due to the associated paste content increase with the increase of the binder content. Koehler [40] confirmed that an increase in paste volume results in an increase in slump, reduction in yield stress, and plastic viscosity. The paste is an essential component of the mixture which coats the sands and fills the spaces between the sands and provides workability. With the increase of the paste content, the distance between the sand particles is increased. The paste acts as a lubricant between the sand particles. This reduces the resistance of the mixture to the shearing applied, hence reducing the yield stress and plastic viscosity. With the decrease of binder content, the sand increases, and the internal friction also increases, resulting in higher viscosity and yield stress.

#### 3.3.5. Effect of SP and Borax 

Both the SP and borax reduced the mixtures’ yield stress and plastic viscosity (Table 3). The addition of 4% SP relative to the total binder content reduced the yield stress by 46%, while only 2% borax reduced it by about 49%, showing that borax is more effective than the SP in reducing the yield stress. Adding both SP and borax reduced the yield stress by about 63%. The plastic viscosity also decreased (by about 25%) with the addition of either or both of the admixtures. 

Previous works of the authors [41] show that borax is an effective retarder for the AAC mixture under investigation. The results from the current study further show that borax can reduce yield stress and plastic viscosity. Furthermore, borax has no negative effect on the strength of AAC mixtures; in fact, it slightly improves the compressive strength [41,42,43]. This is of high importance, as the admixture technology in AAC is still in its early stages. Borax reacts with calcium ions, forming a calcium-based borate layer [42]. This additional layer on the surface of the calcium at the early stage of the reaction could be the cause of both retardation and lower yield stress and viscosity.

#### 3.3.6. Effect of SF 

SF is an extremely fine and spherical powder. The particles can fill the voids between other particles, improve the gradation, increase the packing density, and even create a lubrication effect [44]. The high specific surface area and high chemical activity, compared to FA, could also result in higher water demand and inter-particle friction [45]. As shown in Figure 7, when the SF content increased from 5% to 15%, the yield stress decreased. However, when the SF is further increased to 25%, the yield stress increased back. Up to 15%, the SF improved the fluidity of the mixture, probably due to the lubrication effect. Further increase of the SF may have increased the mixture’s water demand, hence the higher yield stress. The plastic viscosity of the mixes decreased with the increase of the SF content, especially from 5% to 15%. The plastic viscosity decreased despite the decrease in the flow of the mixture as the SF content increased. This shows that the addition of SF is an effective way to reduce the viscosity of AAC mixtures. However, as can be observed in Figure 8, the reduction in viscosity was minor when the SF increased from 15% to 25%. This could be due to the two opposing effects of the SF, i.e., the lubrication and the higher water demand effects. 

Previous studies have also observed a similar effect of SF. Li et al. [10] observed that the addition of SF significantly increased the yield stress and decreased the plastic viscosity of alkali-activated slag/glass powder pastes. Without the SF, Li et al. [10] observed that the mixtures followed the modified Bingham model; however, the relationship significantly changed with the SF, which fitted the Bingham model better. Correa-Yepes et al. [34], in their study on self-compacting OPC concrete with FA and SF, observed similar behavior with the increase of SF and suggested that the higher reactivity and fineness of SF accelerating the formation of flocs is a possible reason for the higher yield stress. Memon et al. [35], in their study on the behavior of FA-based AAC with SF replacement, observed the decrease in slump flow and increase in V-funnel flow time. Slump flow is inversely proportional to the yield stress, while V-funnel flow time is directly proportional to viscosity [46,47,48,49]. This shows the increase in both yield stress and viscosity with the increase of SF.

#### 3.3.7. Thixotropy

**Figure 9:**
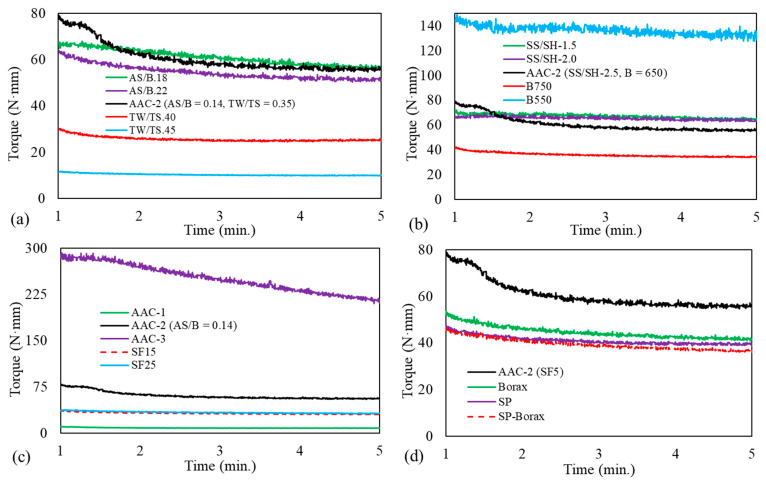
Thixotropic behavior for mix proportion parameters: (**a**) AS/B and TW/TS, (**b**) SS/SH and binder, (**c**) AAC and SF, (**d**) borax and SP.

Figure 6 shows the hysteretic response of the mixtures. The up-ramp was always higher than the down-ramp. The viscosity in the down-ramp is smaller than the up-ramp, showing the time-dependent nature of the mixture’s rheology behavior. Such a response usually means that the material is thixotropic, meaning that its behavior depends on the shear history [50]. Another way to visualize this is by plotting the constant speed region of the profile (i.e., 120 rpm in Figure 4) and studying the change in torque at this constant speed. As shown in Figure 9, most of the mixes showed a decrease in the torque value despite maintaining the constant speed, thus showing the thixotropic nature of the mixture. Understanding concrete thixotropy is essential in modern construction. It influences various aspects of concrete construction, such as formwork pressure, multi-layered castings, and segregation resistance [51].

There are different types of thixotropy and measurement methods [51]. Hysteresis areas may give a primary indication of the thixotropic behavior; however, quantitative determination of thixotropy is not always certain due to variations of hysteresis loops and the coupling of the shear rate and time [51,52]. For a comparative analysis of thixotropy of the mixes in the current study, the difference in area between the up- and down-ramp including the constant region (Figure 6) is used (Table 4). This area is referred as the thixotropic area; it provides a measure of the work required to break the initial linkages and internal friction of the mixtures before equilibrium is attained. As can be observed in Figure 9, after an initial high torque, the torque decayed to a minimum value at the constant speed of 120 rpm. This can be taken as the equilibrium torque that is independent of the shear history.

The maximum thixotropic area was observed for the AAC-3 mixture. AAC-3 has a low TW/TS ratio (0.24 compared to 0.35 for AAC-2), resulting in high resistance to shearing, which, in turn, resulted in the high thixotropic area observed. From the AAC-2 parametric study mixtures (mixes excluding AAC-1 and AAC-3), the maximum was observed for the B550 mixture, which means that lower binder content results in higher thixotropy. A similar result was reported for self-consolidating concrete [53]. Such behavior is attributed to the relative increase in sand volume that can lead to a greater level of internal friction. The other significant factor in the thixotropic area was the TW/TS. The highest TW/TS ratio, TW/TS.45, showed the lowest thixotropic area. The TW/TS ratio was increased by increasing the TW content and keeping the TS content constant. To keep the unit volume, the sand content decreased as TW/TS ratio increased. The decrease in sand content and the increase in TW resulted in a lower thixotropic area due to the lower degree of internal friction resulting in lower initial shear stress. It is well documented that the binder and aggregate contents and water to binder ratio are among the important factors affecting the degree of thixotropy and structural breakdown [54].

From the SS/SH ratio variations, SS/SH ratio of 2.5 showed the highest drop in torque and the highest thixotropic area (Figure 9 and Table 4). Alkali-activated mixes activated with sodium silicate form a primary C-S-H gel at an early age due to the interaction of the silicate ions from the activator and the calcium ions from the GGBS [16]. This gel is broken when the mix is subjected to shearing at a constant rate. Due to the early C-S-H formation, sodium-silicate-activated mixes are also known to have a faster setting time. Their setting time and workability can be increased by lengthening their mixing times [16]. Hence the higher initial torque of the SS/SH 2.5 (higher silicate) mix is due to the early C-S-H formation, while the lower equilibrium torque is due to the constant shearing (lengthening of mixing).

**Figure 10:**
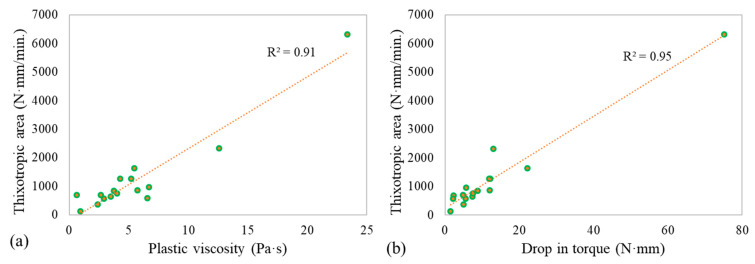
Thixotropic area relation with (**a**) plastic viscosity, (**b**) drop in torque.

The thixotropic area showed good correlation with the viscosity (Figure 10a). The thixotropic nature of the mixtures increased with the increase of viscosity. This is due to the increase of initial torque value with the increase of plastic viscosity. Figure 10b shows the relationship between the drop in torque and thixotropic area. The two methods for thixotropy analysis showed a strong correlation with a high coefficient of correlation (R^2^) value of 0.95. Hence, similar discussions made for the thixotropic area can also be made for the drop in torque.

#### 3.3.8. Rheology and Flowability 

**Figure 11:**
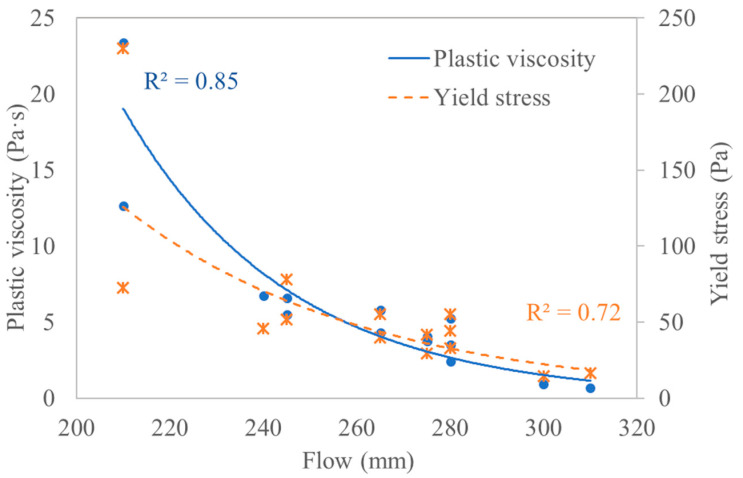
Flow to yield stress and flow to viscosity relationship.

Generally, the workability of cementitious materials shows an inverse relation with yield stress [55]. As can be observed from Figure 11, both the yield stress and plastic viscosity decreased with the increase of the flow values. The viscosity showed a stronger correlation with the flow than the yield stress. Studies on OPC systems showed that the slump is inversely (either linearly, logarithmically, or exponentially) related to the yield stress while the viscosity showed no apparent trends [56]. It is not clear why this difference is observed between AAC and OPC systems in terms of the viscosity-flow relations, and the results of the current study are not enough to give a conclusive remark on the matter. Hence, further research on the topic is recommended.

## 4. Conclusions

Based on the experimental results presented in this study, the following conclusions can be drawn:

- Both yield stress and plastic viscosity decrease with the increase of binder content and water to binder ratio. Binder content has a more substantial effect on both rheology parameters.
- An increase in sodium silicate to sodium hydroxide ratio increases the yield stress. This is due to the formation of early C-S-H products from the reaction of silicate ions from the alkaline solution and calcium ions from the slag.
- Borax is an effective admixture for reducing yield stress. A 2% borax relative to the total binder reduced the yield stress by 49%. This is due to the additional calcium-based borate layer that borax forms at the early stage of the reaction.
- The effect of silica fume on rheology depends on its dosage. Up to 15% replacement of the binder, silica fume decreased the yield stress of the mix due to its lubrication effect; however, further increase resulted in higher water demand and hence higher yield stress.
- Plastic viscosity decreases with the increase of silica fume. This was observed despite the decrease in the flow value when silica fume increased, showing that addition of silica fume is an effective way to reduce viscosity.
- Thixotropic area significantly increases with the decrease in binder content, and decreases with the increase in water content.

## Figures and Tables

**Table 1:** Chemical composition of ingredients [27].

Composition	FA (%)	GGBS (%)	SF * (%)
SiO_2_	49.79	34.48	93.81
Al_2_O_3_	26.71	11.48	0.48
Fe_2_O_3_	8.57	-	1.49
MgO	2.47	7.08	0.46
CaO	4.34	42.43	0.30
K_2_O	3.36	0.66	0.77
Na_2_O	1.28	0.56	0.42
SO_3_	1.49	2.17	0.20
TiO_2_	1.23	1.14	-
Specific surface area (m^2^/g)	0.45	0.46	19.40
Specific gravity (g/cm^3^)	2.28	2.91	2.20

* Manufacturer’s specification.

**Table 2:** Mix proportions.

Mix No.	Binder (kg/m^3^)	Sand (kg/m^3^)	AS/B	SS/SH	TW/TS	SP (%)	Borax (%)
AAC-1	550	1104	0.14	2.5	0.55	-	-
AAC-2	650	1159	0.14	2.5	0.35	-	-
AAC-3	750	1195	0.18	2.5	0.25	-	-
TW/TS.40	650	1062	0.14	2.5	0.40	-	-
TW/TS.45	650	966	0.14	2.5	0.45	-	-
SS/SH-1.5	650	1157	0.14	1.5	0.35	-	-
SS/SH-2.0	650	1158	0.14	2.0	0.35	-	-
AS/B.18	650	1117	0.18	2.5	0.35	-	-
AS/B.22	650	1074	0.22	2.5	0.35	-	-
B750	750	941	0.14	2.5	0.35	-	-
B550	550	1377	0.14	2.5	0.35	-	-
SP-Borax	650	1159	0.14	2.5	0.35	4	2
SP	650	1159	0.14	2.5	0.35	4	-
Borax	650	1159	0.14	2.5	0.35	-	2
SF15	SF (15)	1159	0.14	0.14	2.5	0.35	-
SF25	SF (25)	1159	0.14	0.14	2.5	0.35	-

Binder: 55% FA, 40% GGBS, and 5% SF. AS: the solid part of the alkaline solution, TS: total content of the binder solids (binder + AS), TW: total water, SS and SH: solid parts of sodium silicate and sodium hydroxide.

**Table 3:** Flow, yield stress, and plastic viscosity parameters.

Mix No.	Flow (mm)	Yield Torque (N·mm)	Viscosity (N·mm·min)	Yield Stress (Pa) [31]	Viscosity (Pa·s) [31]
AAC-1	310	1.55	0.05	16.48	0.67
AAC-2	245	7.35	0.41	78.13	5.51
AAC-3	210	6.84	1.74	72.71	23.36
TW/TS.4	280	4.18	0.18	44.43	2.42
TW/TS.45	300	1.36	0.07	14.46	0.94
SS/SH-1.5	240	4.32	0.50	45.92	6.71
SS/SH-2	245	4.87	0.49	51.77	6.58
AS/B.18	265	5.19	0.43	55.17	5.77
AS/B.22	280	5.17	0.39	54.96	5.24
B750	280	3.08	0.26	32.74	3.49
B550	210	21.64	0.94	230.03	12.62
SP-Borax	275	2.74	0.28	29.13	3.76
SP	275	3.93	0.30	41.78	4.03
Borax	265	3.73	0.32	39.65	4.30
SF15	200	4.49	0.22	47.73	2.95
SF25	145	7.74	0.20	82.28	2.69

**Table 4:** Thixotropy.

Mix	Drop in Torque (N·mm)	Thixotropic Area (N·mm/min.)
AAC-1	2.4	677
AAC-2	22.3	1624
AAC-3	75.2	6314
TW/TS.4	5.0	354
TW/TS.45	1.5	124
SS/SH-1.5	5.7	957
SS/SH-2	2.3	571
AS/B.18	12.1	849
AS/B.22	12.2	1252
B750	7.5	632
B550	13.1	2314
SP-Borax	8.9	838
SP	7.5	740
Borax	12.0	1258
SF15	5.6	557
SF25	4.9	694

## Data Availability

The data presented in this study are available on request from the corresponding author.
